# Changes in hormone receptor when breast cancer metastasizes to the colon: case report and literature review

**DOI:** 10.3389/fonc.2024.1391393

**Published:** 2025-01-29

**Authors:** Huimeng Li, Liying Yang, Xiqiang Sun, Zhuquan Wang, Shuangwei Qin, Chengcheng Li, Gongwu Liu, Fengming Xie, Weiwei Gao

**Affiliations:** ^1^ Department of Thyroid and Breast Surgery, Southern Central Hospital of Yunnan Province, The First People’s Hospital of Honghe State, Honghe Hospital Affiliated to Kunming Medical University, Gejiu, Yunnan, China; ^2^ Department of Pathology, Southern Central Hospital of Yunnan Province, The First People’s Hospital of Honghe State, Honghe Hospital Affiliated to Kunming Medical University, Gejiu, Yunnan, China; ^3^ Department of Hepatobiliary Surgery, Southern Central Hospital of Yunnan Province, The First People’s Hospital of Honghe State, Honghe Hospital Affiliated to Kunming Medical University, Gejiu, Yunnan, China; ^4^ Department of Rheumatology and Immunology, Southern Central Hospital of Yunnan Province, The First People’s Hospital of Honghe state, Honghe Hospital Affiliated to Kunming Medical University, Gejiu, Yunnan, China

**Keywords:** colon metastasis, progesterone receptor, estrogen receptor, non-specific types of breast cancer, breast cancer

## Abstract

The metastasis of breast cancer to the colon is a rare occurrence, especially in the presence of changes in estrogen and progesterone receptors. To date, literature has only reported two cases of invasive ductal carcinoma and two cases of invasive lobular carcinoma metastasizing to the colon with concurrent changes in hormone receptors. This report describes a 65-year-old woman with a history of left breast cancer, who presented with symptoms of bloody stools and abdominal pain. CT and colonoscopy results revealed a malignant tumor in the ascending colon, and the patient underwent surgery. Pathological results post-surgery indicated changes in hormone receptors, differing from the previous breast cancer pathology, ultimately leading to the diagnosis of breast cancer metastasis to the colon. The patient was found to have liver metastasis 14 months after right hemicolectomy, and systemic metastases in various locations were discovered at the 19-month mark.

## Introduction

Breast cancer is the most common malignant tumor in women. Recurrence and distant metastasis often pose challenges in treatment. Common sites of metastasis include bones, liver, and lungs, while gastrointestinal metastases are extremely rare. Previous reports have shown that the pattern of metastasis differs between lobular carcinoma and ductal carcinoma of the breast. Gastrointestinal, gynecologic, and peritoneal metastases are more common in lobular carcinoma (1, 2). ILC (invasive lobular carcinoma) has an increased tendency to metastasize to the GI (gastrointestinal) tract compared with breast carcinomas of NST (non-special type) (40% vs. 2%) (3). We report a case of infiltrating ductal carcinoma that metastasized to the ascending colon, with changes in estrogen receptor (ER), progesterone receptor (PR), and human epidermal growth factor receptor-2 (HER2) status. To our knowledge, there have been only two similar cases reported previously.

## Case presentation

**Figure 1:**
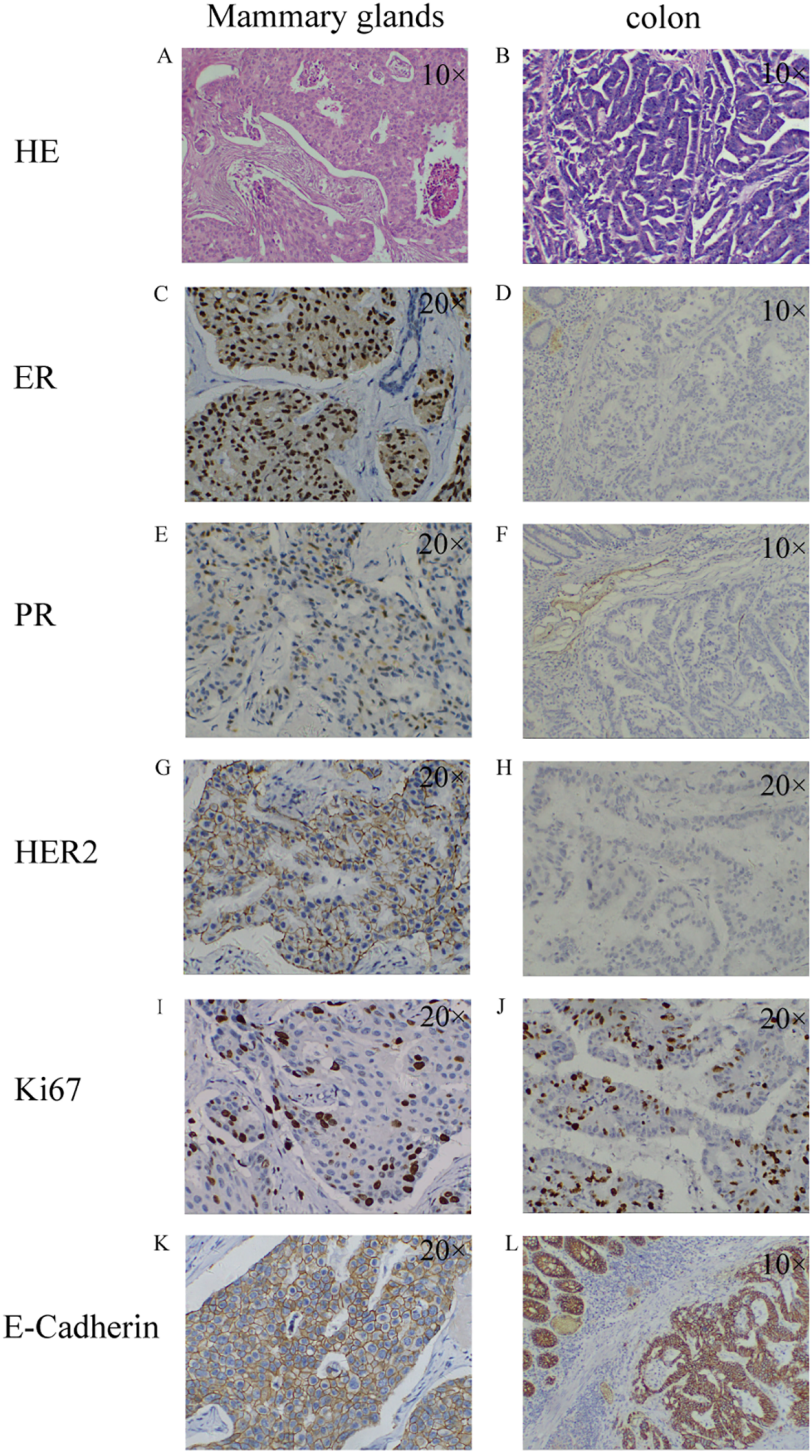
Pathological findings of breast malignancy and colon malignancy. breast malignancy: HE staining **(A)**; ER+ **(C)**; PR+ **(E)**; HER2+ **(G)**; Ki67 15%+ **(I)**; E-Cadherin+ **(K)**; colon malignancy: HE staining **(B)**; ER- **(D)**; PR- **(F)**; HER2- **(H)**; Ki67 50%+ **(J)**; E-Cadherin+ **(L)**. HE staining: Hematoxylin-Eosin staining; ER: estrogen receptor; PR: progesterone receptor; HER2: human epidermal growth factor receptor-2; Ki67: nuclear-associated antigen Ki67.

A 65-year-old woman presented to our center with intermittent abdominal pain and hematochezia for several months. She had a previous diagnosis of ER- and PR-positive (**Figure 1**), HER2 ++ (no further testing was done) invasive ductal carcinoma of the left breast 5 years before. She underwent modified radical mastectomy for breast cancer. The pathological examination did not indicate the presence of axillary lymph node metastasis and received chemotherapy with anthracycline and cyclophosphamide, followed by taxanes. Subsequently, she received 5 years of endocrine therapy with letrozole until she presented to our center. The patient denied having smoked or consumed alcohol in the past.

**Figure 2:**
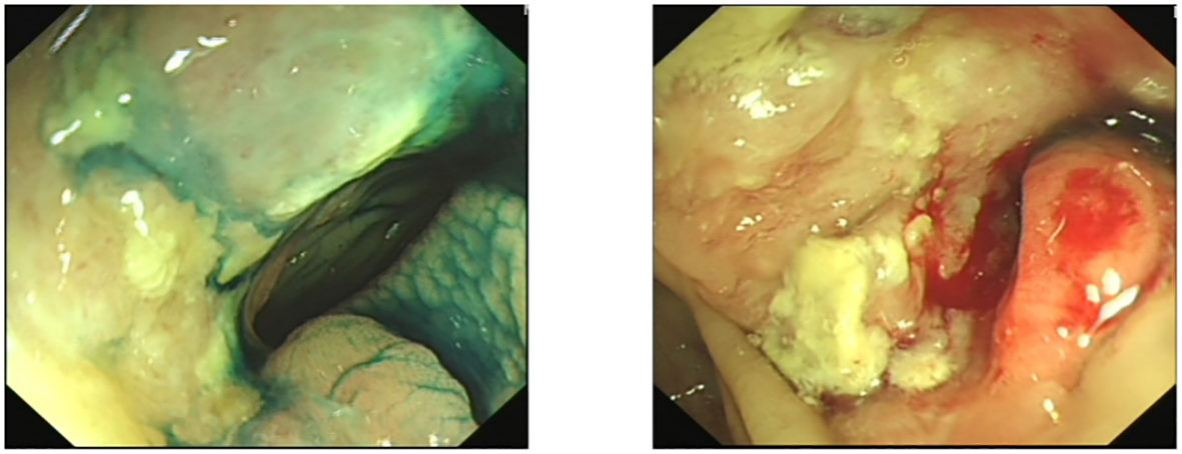
Colonoscopy revealed a mass of ascending colon.

During colonoscopy, a mass was found in the ascending colon (**Figure 2**). Biopsy results confirmed metastasis of breast cancer to the colon, which was negative for ER, PR, and HER2. However, no local lesions or enlarged lymph nodes were detected in the contralateral breast, chest wall, or axilla through ultrasound, magnetic resonance imaging, and clinical palpation.

**Figure 3:**
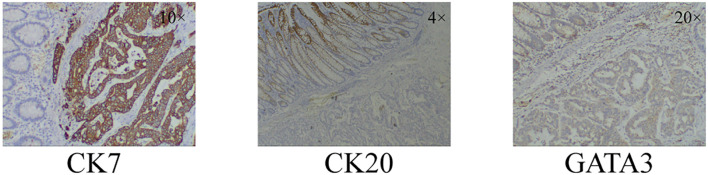
Immunohistochemical staining results of colonic malignancies. CK7+, CK20-, GATA3+. CK7: Cytokeratin 7; CK20: Cytokeratin 20; GATA3: GATA 3 binding protein.

After receiving a transfusion of six units of red blood cells to correct anemia, the patient underwent a right hemicolectomy. Intraoperatively, it was observed that the tumor had invaded the right renal fascia. Two lymph node metastases were found on pathological examination after palliative surgery for colonic malignancy. The postoperative pathological examination confirmed the colonoscopy biopsy findings. Further immunohistochemical staining revealed positive results for Cytokeratin 7 (CK7), negative results for Cytokeratin 20 (CK20), and positive results for GATA 3 binding protein (GATA3) and E-Cadherin (**Figure 3**).

Considering the rarity of this case, we sought opinions from pathologists in different centers to confirm the diagnosis. After considering the majority of expert opinions, it was confirmed as breast cancer metastasis to the colon. The patient was prescribed oral capecitabine at a dosage of 650-mg/m^2^ twice daily for chemotherapy treatment.

**Figure 4:**
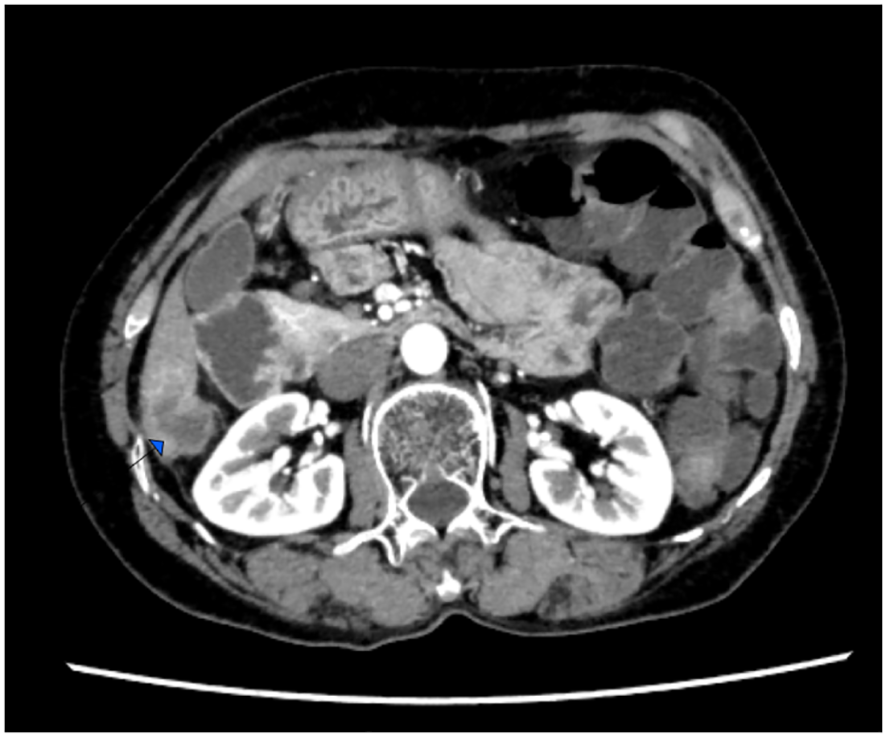
Computedtomography (CT) scan, a solitary nodule was detected in the liver, indicating a metastatic lesion.

**Figure 5:**
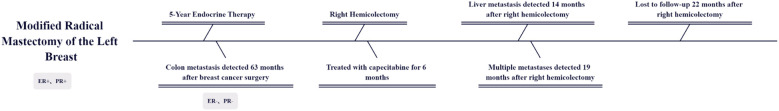
Timeline depicting the treatment and follow-up of the patient.

Follow-up imaging studies performed 6 months after the operation did not show any tumor metastasis or recurrence. However, the patient discontinued medication due to severe hand–foot syndrome. In a 14-month postoperative computed tomography (CT) scan, a solitary nodule was detected in the liver, indicating a metastatic lesion (**Figure 4**). The patient refused intravenous chemotherapy and opted for a second course of oral capecitabine treatment. Nineteen months after surgery, multiple metastases were discovered, including the liver, abdominal lymph nodes, adrenal glands, thoracolumbar spine, lungs, and mediastinal lymph nodes, accompanied by widespread cancer-related pain. The patient declined medication treatment. During the entire follow-up, no local recurrence of breast malignancy or new tumors of the opposite breast were found. The patient was lost to follow-up at 20 months after right hemicolectomy. Treatment and follow-up are organized into a timeline (**Figure 5**).

## Discussion

Breast cancer metastases to the gastrointestinal tract is extremely rare. Previous reports have shown that the most common sites of metastases in breast cancer are the bones, lungs, liver, and brain, while gastrointestinal metastases, especially to the colon, is very rare. The incidence of breast cancer metastases to the gastrointestinal tract, particularly to the colon, is estimated to be around 1%, with a rate of approximately 0.1% specifically for metastases to the colon (4). The patterns of metastases differ between lobular carcinoma and ductal carcinoma of the breast, with gastrointestinal metastases being more common in lobular carcinoma (1, 2). In this case, it was the ductal carcinoma of the breast that had colon metastases.

Due to the final diagnosis of breast cancer metastasis to the colon with receptor changes, this case presents significant challenges in terms of diagnosis and treatment. We performed a search in the PubMed database and gathered relevant literature, as detailed in **Table 1**. Our findings reveal the fourth documented case of ductal carcinoma of breast metastasizing to the colon, accompanied by changes in receptor status. Additionally, there have been two cases where receptor changes were observed when lobular carcinoma metastasized to the colon (5, 6).

**Table 1:** Case reports of breast carcinomas with colonic metastases.

Author	Year	Age	Primary(breast)			Treatment	Time since first diagnosis (years)	Metastasis	
			Site	Pathological types	Biomarkers			Site	Biomarkers
Takeuchi H (18)	2012	38	left	ILC	ER+,PR-,HER2-	Surgery, Chemotherapy, Radiation therapy, Endocrine Therapy	3	Stomach and Colon	ER+,PR-, HER2- -
Jansen van Rensburg A (19)	2021	74	left	DC	ER+,PR-	Surgery, Radiation therapy, Endocrine Therapy	27	Bone,ovary, sigmoid colon	ER +, PR+ E-cadherin+, GATA3+,CK20-
Sheen-Chen SM (20)	2008	41	right	IDC	–	–	Simultaneous	ovary and omentum	- CK7+、CEA+、CK20-
Higley C (21)	2020	74	right	–	–	breast-conserving surgery and radiation therapy, Endocrine Therapy	40	transverse colon.	ER-、PR-、HER2- CDX-2-、CK20-、GATA3+、E-cadherin-、PD-L1-
Khan I (22)	2017	56	right	ring cell adenocarcinoma	–	–	Simultaneous	stomach, small intestine, and colon	–
Noor A (23)	2020	68	–	ILC	ER+,PR+,HER2-	Surgery, Radiation therapy, Endocrine Therapy	30	Bone、sigmoid colon	ER+、PR+、HER2-,CK7+, CK20-,CDX2-,CA19-9-
Bering J (5)	2020	67	left	ILC	ER+,PR+,HER2-	Endocrine Therapy	Simultaneous	transverse colon	ER-,PR-,HER2+ GATA3+,CK7+,CDX2-,CK20-,E-cadherin-,GCDFP-15+
Abid A (24)	2013	59	left	LC	ER+,PR+,HER2-	–	Simultaneous	stomach, duodenum, and colon	ER+、PR+ GCDFP-15+
Takedomi H (4)	2019	76	right	ILC	–	Chemotherapy	Simultaneous	descending colon	- GCDFP-15+,mammaglobin+
Abu Zaanona MI (25)	2020	73	–	–	–	Endocrine Therapy	–	colon	ER+,PR-,HER2-,Pancytokeratin+, GATA3 +, E-cadherin-, CDX2-, SOX10-
Inoue H (26)	2022	63	right			Chemotherapy,Endocrine Therapy	15		ER+,PR-,HER2+,CK7+, GATA3+, ER+, HER2+, CK20-, PR-, E-cadherin-, GCDFP15- ,caudal-related homeobox 2
Mostafa A (27)	2002	56	–	IDC	ER+,PR+	Surgery, Radiation therapy, Endocrine Therapy	5	descending colon	ER-,PR--
Tsujimura K (28)	2017	51	left	ILC	ER+,PR+	–	Simultaneous	ileocecal	ER+,PR+,HER2-
Feng CL (29)	2009	49	right	IDC	–	–	2	Colon	CK7+, CK20-
Kobayashi M (30)	2020	74	–	ILC	–	surgery	23	stomach and colon	CAM5.2+,ER+, E-cadherin-
Théraux J (31)	2008	69	bilateral	IDC	–	Surgery, Endocrine Therapy	28	transverse colon	ER+,PR+, HER2-, CK7+, CK20-
Malhotra A (32)	2009	71	–	–	–	–	–	bone, transverse colon	–
Villa Guzmán JC (33)	2017	58	–	ILC	ER+,PR+,HER2-	Surgery, Chemotherapy, Radiation therapy, Endocrine Therapy	3	stomach	cytokeratin-19+,ER+
Gerova VA (34)	2012	56	left	ILC	PR+	Surgery, Chemotherapy, Radiation therapy, Endocrine Therapy	5	stenotic, stomach	–
		42	left	–	ER+,PR+	Surgery, Chemotherapy, Radiation therapy, Endocrine Therapy	7	stomach	ER+,PR+,CK7+, E-cadherin+,S-100+, GCFP15-,CDX2-
Wang G (35)	2014	70	left	IDC	E-cadherin+、34βE12-、ER+、PR+、CK19+、CK20+	Surgery, Radiation therapy, Endocrine Therapy	10	ascending colon	CK7+、E-cadherin+、34βE12-、ER+、GCDFP-15+、CK19+、CK20+
Cho DH (36)	2011	46	bilateral	IDC	ER+,PR+,HER2-	Surgery, Chemotherapy, Radiation therapy, Endocrine Therapy	2	terminal ileum	ER+,PR-,HER2+,
Algethami NE (37)	2022	47	bilateral	ILC	ER+,PR+,HER2-	Surgery, Radiation therapy, Endocrine Therapy	4	rectum	ER+、HER2+、Pan-cytokeratin +
Zhou XC (38)	2012	54	right	IDC	ER+,PR+,P53+, HER2-	Surgery, Chemotherapy, Radiation therapy, Endocrine Therapy	8	sigmoid colon	CDX2-, Villin-, TTF-1-, -CK20-, HER2-, ER- ,PR-, CK7+,p53+
Andriola V (39)	2014	63	left	IDC,LC	–	Surgery, Chemotherapy, Radiation therapy, Endocrine Therapy	23	colon and terminal ileum	CK19+, GCFDP-15+,HER2+
Motos-Micó J (40)	2014	69	right	ILC	–	Surgery, Chemotherapy	18	sigmoid colon	ER-,PR-,HER2-,CK7+
Abdallah H (41)	2020	59	right	ILC	ER+,PR+,HER2-	–	Simultaneous	endometrium, myometrium, fibroid and cervix	CK7+,GATA3+,ER+,PR+, desmin-, CD10-, actin- ,Caldesmon-
		66	right	ILC	CK+,ER+,PR+,HER2-	–	Simultaneous	Colon	CK7+,GATA-3+,ER+,CK20-,CDX2-
		53	right	ILC	–	–	Simultaneous	intestine, omentum, and peritoneal wall, bilateral ovarian, stomach	CK7+、GATA-3+,ER+,E-cadherin+,CK20-,CDX2-
Michalopoulos A (42)	2004	55	left	IDC	–	Surgery, Chemotherapy	4	transverse colon	CK7+,milk-fat globule protein+,
		57	left	ILC	–	Surgery, Chemotherapy, Radiation therapy	10	transverse colon	CK7+,milk-fat globule protein+, CEA+,ER+, weakly positive for cytokeratin 20 and Breast II.
Schellenberg AE (43)	2018	69	left	IDC	E-cadherin+,ER+,PR+,HER2+	Surgery, Chemotherapy, Radiation therapy, Endocrine Therapy	2	rectosigmoid	CK-7+, E-cadherin+, GCDFP+, mammoglobin+,ER+,PR+,CK-20-,CDX-2--
Matsuda I (44)	2012	62	left	ILC	–	Surgery	24	ascending colon and rectum	CK7+,ER+,CK20-,E-cadherin-
Koleilat I (45)	2010	54	right	IDC	–	Surgery, Chemotherapy, Radiation therapy, Endocrine Therapy	13	colon	ER+,PR+,HER2-
Mistrangelo M (46)	2011	80	left	ILC	–	Surgery, Endocrine Therapy	25	sigmoid colon	–
Razzetta F (47)	2011	77	bilateral	ILC(left),IDC(right)	–	–	Simultaneous	right, transverse and left colon	ER+,PR-
Cervi G (48)	2001	59	–	ILC	–	Surgery	8	rectum	ER+,PR+
Amin AA (49)	2011	61	right	ILC	–	Surgery, Endocrine Therapy	17	rectum	CK 20-,ER+,CK7+,PR+
López Deogracias M (6)	2009	67	left	ILC	ER+,PR+	Surgery	15	rectum	ER-,PR-,CK20-,CDX2-
Law WL (50)	2003	49	left	IDC	ER+	Surgery, Endocrine Therapy	5	descending colon	ER+
Samra B (51)	2019	64	–	–	–	–	–	colon,right sacral iliac	CK-7+,GATA-3+,ER+,MOC31+,CK-20-,CD-X2-,PR-,PAX-8-,SOX-10-,CD4-5,chromogranin-, synaptophysin-, TTF-1-
Kim HW (52)	2014	46	right	metaplastic	ER-,PR-,HER2-	Surgery, Chemotherapy, Radiation therapy	2	sigmoid colon	CK20-,CK5/6-,ER-,PR-,HER2-,CK7+
Signorelli C (53)	2005	62	right	ILC	–	Surgery	12	right chest wall, colon	ER+,PR+,P53-,HER2-
Gizzi G (54)	2015	72	–	–	ER+,PR+	Surgery, Chemotherapy, Radiation therapy, Endocrine Therapy	11	sigmoid colon	CK7+,GATA3+, ER+,PR+,HER2-
Dhar S (55)	2003	75	left	–	ER+	Surgery	6	sigmoid colon	ER+,CK7+,CK20-
Maekawa H (56)	2012	52	right	IDC	ER+,PR+	Surgery, Chemotherapy, Radiation therapy, Endocrine Therapy	16	ascending colon	–
Koufopoulos N (57)	2018	87	–	–	–	–	–	Colonic serosa	CK-20-,CDX-2-,CK7-,r GATA-3+, mammaglogin+,ER+,PR+, E-cadherin-, Chromogranin-,synaptophysin-
Critchley AC (58)	2011	62	–	IDC	–	Surgery, Chemotherapy, Radiation therapy, Endocrine Therapy	8	stomach, ascending colon	ER+,PR+,HER2-,CK7+,CK20-
Kachi A (59)	2019	58	left	ILC	–	Surgery, Chemotherapy, Radiation therapy, Endocrine Therapy	6	sigmoid colon, appendix, and ovaries.	ER+,PR+
Blachman-Braun R (60)	2018	73	bilateral	–	–	Surgery, Chemotherapy,	15	colon	ER+,PR-,HER2-
Jia J (61)	2023	67	right	IDC	ER+,PR+,HER2-	Surgery, Chemotherapy, Endocrine Therapy	10	Appendix, colon	GATA-3+,GCDFP-15+,ER+,PR+,HER2+, E-cadherin+,p120+,CK7+,CK20-,SATB-2-,Villin-,syn-,CgA-,CD56-
Arif FZ (62)	2023	65	bilateral	IDC	triple-negative (left), HER2+(right)	–	–	descending colon	–

ILC, Invasive lobular carcinoma; IDC, Invasive ductal carcinoma; DC, ductal carcinoma; LC, lobular carcinoma; ER, estrogen receptor; PR, progesterone receptor; HER2, human epidermal growth factor receptor-2; CK7, Cytokeratin 7; CK20, Cytokeratin 20; GATA3, GATA 3 binding protein. CEA, Carcinoembryonic antigen; CDX2, caudal-related homeobox transcription factor 2; GCDFP, Gross cystic disease fluid protein; CAM5.2, cytokeratin CAM5.2; S-100, closely related, small, acidic Ca2+-binding proteins; 34βE12, cytokeratin 34βE12; P53, tumour suppressor gene p53; PAX-8, a nephric cell lineage transcription factor; SOX-10, SRY-related HMG-box 10; CD45, receptor-like protein tyrosine phosphatase; TTF-1, Thyroid transcription factor 1; CK5/6, cytokeratin 5/6; p120, p120-catenin; SATB-2, special AT-rich sequence-binding protein 2; syn, Synuclein; CgA, Chlorogenic acid; CD56, Neural cell adhesion molecule; SOX10, transcription factors SOX10.

In this case, there was a mismatch between the receptors in the primary breast lesion and the colon metastases. Incompatible receptors between the primary and secondary lesions of breast cancer have been reported, with frequencies of alterations in ER, PR, and HER2 being approximately 16.4%–32.4%, 30.9%–37.78%, and 10.2%–14.5%, respectively (7–10). Emilia Montagna reported in 2017 that approximately 82% of breast cancer metastases in the gastrointestinal tract have positive hormone receptor expression (11). In the dozens of case reports we have collected, the majority of patients were found to be hormone receptor positive. Among these patients, most did not exhibit any changes in the hormone receptor status. Previous reports have indicated that the instability of hormone receptors in breast cancer is associated with a poorer prognosis (12, 13), Women whose ER-positive primary tumors transform into ER-negative tumors experience a significant 48% increase in the risk of death (7). In this case report, liver metastases were found at 14 months after surgery, and multiple metastases throughout the body were found at 19 months with subsequent rapid disease progression.

CK7, CK20, and GATA3 are commonly used in pathology for tumor diagnosis and classification. These markers can provide information about the type and origin of the tumor. CK7 was expressed in 89%–98% of non-specified breast cancers (14). Most gastrointestinal, pancreaticobiliary, and ovarian mucinous adenocarcinomas are CK20 positivity, in adenocarcinoma, positivity of CK20 strongly indicates a non-breast origin, and CK7−/CK20+ immunoprofile strongly suggests colorectal origin (15). Based on previous research reports, CK7+/CK20 should be considered indicative of a tumor originating from the breast. GATA3 is also thought to be often positive in primary breast disease (16, 17). It was based on the immunohistochemical staining results of CK7 positive, CK20 negative, and GATA3 positive, combined with the opinions of multicenter pathologists, showing that the case was finally diagnosed as breast cancer with colon metastasis. Even though this case is very rare, we should still pay attention to the occurrence of colon metastasis in the diagnosis and treatment of breast cancer.

## Conclusions

This case serves as a reminder to clinicians that they should consider rare sites of metastases and different receptor expression patterns in breast cancer patients in order to make more accurate treatment decisions. However, further research is needed to explore the mechanisms of this metastatic pattern and related treatment strategies.

## Data Availability

The datasets presented in this article are not readily available due to participant privacy. Requests to access the datasets should be directed to the corresponding authors.
